# Combining rTMS With Intensive Language-Action Therapy in Chronic Aphasia: A Randomized Controlled Trial

**DOI:** 10.3389/fnins.2018.01036

**Published:** 2019-02-04

**Authors:** Paula H. Heikkinen, Friedemann Pulvermüller, Jyrki P. Mäkelä, Risto J. Ilmoniemi, Pantelis Lioumis, Teija Kujala, Riitta-Leena Manninen, Antti Ahvenainen, Anu Klippi

**Affiliations:** ^1^Department of Psychology and Logopedics, Faculty of Medicine, University of Helsinki, Helsinki, Finland; ^2^Brain Language Laboratory, Department of Philosophy and Humanities, WE4, Freie Universität Berlin, Berlin, Germany; ^3^BioMag Laboratory, HUS Medical Imaging Center University of Helsinki and Helsinki University Hospital, Helsinki, Finland; ^4^Department of Neuroscience and Biomedical Engineering, Aalto University School of Science, Espoo, Finland; ^5^Temerty Centre for Therapeutic Brain Intervention, Centre for Addiction and Mental Health, and Department of Psychiatry, University of Toronto, Toronto, ON, Canada; ^6^Cognitive Brain Research Unit, Department of Psychology and Logopedics, Faculty of Medicine, University of Helsinki, Helsinki, Finland

**Keywords:** chronic aphasia, language rehabilitation, efficacy, ILAT, rTMS, randomized controlled trial (RCT)

## Abstract

Neuromodulation technologies, such as transcranial magnetic stimulation (TMS), are promising tools for neurorehabilitation, aphasia therapy included, but not yet in common clinical use. Combined with behavioral techniques, in particular treatment-efficient *Intensive Language-Action Therapy* (ILAT, previously CIAT or CILT), TMS could substantially amplify the beneficial effect of such behavioral therapy alone (Thiel et al., 2013; Martin et al., 2014; Mendoza et al., 2016; Kapoor, 2017). In this randomized study of 17 subjects with post-stroke aphasia in the chronic stage, we studied the combined effect of ILAT and 1-Hz placebo-controlled navigated repetitive TMS (rTMS) to the right-hemispheric inferior frontal cortex—that is, to the anterior part of the non-dominant hemisphere's homolog Broca's area (pars triangularis). Patients were randomized to groups A and B. Patients in group A received a 2-week period of rTMS during naming training where they named pictures displayed on the screen once every 10 s, followed by 2 weeks of rTMS and naming combined with ILAT. Patients in group B received the same behavioral therapy but TMS was replaced by sham stimulation. The primary outcome measures for changes in language performance were the Western Aphasia Battery's aphasia quotient AQ; the secondary outcome measures were the Boston naming test (BNT) and the Action naming test (Action BNT, ANT). All subjects completed the study. At baseline, no statistically significant group differences were discovered for age, post-stroke time or diagnosis. ILAT was associated with significant improvement across groups, as documented by both primary and secondary outcome measures. No significant effect of rTMS could be documented. Our results agree with previous results proving ILAT's ability to improve language in patients with chronic aphasia. In contrast with earlier claims, however, a beneficial effect of rTMS in chronic post-stroke aphasia rehabilitation was not detected in this study.

**Clinical Trial Registration:** www.ClinicalTrials.gov, identifier: NCT03629665

## Introduction

Language involves a complex neuronal network distributed across the brain. There is a general consensus on the dominance of the left hemisphere for most elements of language processing (Somers et al., 2015). The right hemisphere contributes particularly in expressive and receptive language (Kertesz, 1985). Lesions of the left hemisphere disturb language functions more severely than those of the right one (Pedersen et al., 1995). Hemispheric transfer of linguistic functions to the non-dominant side may be one of the mechanisms of recovery in aphasia (Pulvermüller and Schönle, 1993; Saur et al., 2006; Saur and Hartwigsen, 2012; Turkeltaub, 2015). However, in a different perspective, the right non-dominant hemisphere is seen as a hindrance rather than a supplement of language recovery. Accordingly, activity in the right-hemispheric homologs of the perisylvian language areas may interfere with the left-hemispheric language mechanisms and reduce performance. One strategy of language therapy has therefore aimed at inhibiting these right-hemisphere homologs. Particularly the anterior part of the right hemisphere's Broca's homolog area (BA 45) has been investigated intensively; previous work indicates that inhibitory repetitive TMS to this site may improve the outcome of aphasia rehabilitation (Turkeltaub, 2015).

Intensive language use influences the reorganization and functional restitution of language networks in post-stroke aphasia (Berthier and Pulvermüller, 2011; Brady et al., 2016), with important implications for rehabilitation. New behavioral treatment approaches emphasize massed practice in a short time, thus maximizing therapy quantity and frequency and, therefore, the correlation of the behavioral and neuronal changes (Kleim, 2011). A relatively novel approach in post-stroke aphasia, *Intensive Language-Action Therapy* (ILAT, previously CIAT or CILT; e.g., Pulvermüller and Berthier, 2008) is based on three main principles: (i) massed practice, with 3 h of exercise per day for 2 weeks (totaling 30 h), (ii) behavioral relevance, meaning that language is practiced in social-communicative action contexts, (iii) focusing, which emphasizes the tailoring of treatment to the patients' communicative and linguistics abilities and needs, so that any “learned non-use” of language can be overcome. Behavioral therapy is delivered intensively in a small group setting, by way of communicative interaction games approximating everyday linguistic activities. Previous randomized controlled trials (RCTs) of ILAT have demonstrated its effectiveness in chronic post-stroke aphasia (Pulvermüller et al., 2001; Meinzer et al., 2005; for review, see Meinzer et al., 2012), and even suggested that it may be more efficient than other intensive but more conventional methods of aphasia therapy (Pulvermüller et al., 2001; Stahl et al., 2016).

Brain stimulation methods may offer clinically usable methods for language rehabilitation. In particular, transcranial magnetic stimulation (TMS) has been proposed to enhance neurorehabilitation. TMS-induced electric currents in the brain cause depolarization of neurons and consequent action potentials (Platz and Rothwell, 2010). Repetitive TMS stimulation (rTMS) leads to lasting effects on excitability and changes in synaptic connections. Case studies suggest that 1-Hz rTMS to the anterior part of the right hemisphere's Broca-area homolog (pars triangularis) may induce language recovery in chronic post-stroke aphasia by modulating activity in the distributed bi-hemispheric language network (Naeser et al., 2005; Martin et al., 2009a,b; Hamilton et al., 2010; Mendoza et al., 2016; Kapoor, 2017). Recently, Barwood et al. (2011) performed a small-scale RCT, comparing 6 chronic post-stroke aphasia patients treated with 1-Hz rTMS 20 min per day for 10 days with an equally-sized sample receiving sham stimulation. They found no evidence for rTMS-related improvements 1 week after stimulation, but relatively better outcomes 2 months after treatment and, later, particularly in picture naming and description (Barwood et al., 2013). Investigating subacute aphasia patients during the first months after stroke, Thiel et al. (2013) found in a further RCT (*n* = 24) that 1-Hz rTMS (a 10-day protocol of 20 min rTMS) to right pars triangularis improved language more than rTMS at the vertex, their “sham” condition (Thiel et al., 2013). These results led to suggestions that the right hemispheric homolog of Broca's area may somewhat work against the efficient neuroplasticity of language in subacute post-stroke aphasia (Naeser et al., 2005; Martin et al., 2009a,b; Hartwigsen et al., 2013; Kapoor, 2017). With 1-Hz rTMS, neuronal activity in the anterior part of the right pars triangularis, which would normally inhibit its left homolog, is reduced. This could facilitate (or disinhibit) neuronal function and reorganization in the left hemisphere (Naeser et al., 2005; Martin et al., 2009a,b; Hamilton et al., 2011). Some researchers have proposed a different perspective on the role of right hemispheric areas in language recovery. In particular, Saur et al. (2006) and Turkeltaub (2015) hypothesized that right hemisphere activation may be beneficial in the initial stages of recovery, although left-hemisphere activation would grow over time and the right hemisphere's role would therefore diminish. A range of data support a positive role of the right non-dominant hemisphere in the neuroplasticity of language (Pulvermüller and Schönle, 1993; Mohr et al., 2014; Abel et al., 2015; Mohr, 2017).

It has also been suggested that the right non-dominant hemisphere could support rather than hinder language recovery in aphasia. Indeed, some authors (Vines et al., 2011; Al-Janabi et al., 2014) have argued that if the role of the right hemisphere is compensatory it could be useful to strengthen that compensatory role even further by applying excitatory stimulation to the right hemisphere. Vines et al. (2011) applied anodal transcranial direct current stimulation (tDCS) to the posterior inferior frontal gyrus of the right hemisphere to augment the benefits of melodic intonation therapy (MIT) for recovery of six non-fluent aphasic patients. According to them, three therapy sessions led to significant improvements in the fluency of speech. The authors, however, considered these results of six patients as preliminary. Al-Janabi et al. (2014) treated two non-fluent aphasic participants with a combination of an excitatory rTMS (intermittent theta burst stimulation) and MIT. The verbal fluency of one participant improved and the authors argued that the combination of rTMS and MIT might have the potential to improve speech and language recovery.

The frequency of magnetic pulses determines the nature of plastic changes, low frequency stimulation (about 1-Hz) typically being used for suppressing cortical activity and high frequency (5-Hz or more) for increasing cortical excitability (Rossi et al., 2009). The type, duration and intensity of speech language therapy following the stimulation varies across studies. Most studies of rTMS in patients with aphasia have used 1-Hz rTMS to the right pars triangularis, which is homotopic to the anterior part of the Broca area (Mendoza et al., 2016). We chose this most common treatment regime which has also had the most solid results according to many RCT studies (e.g., Mendoza et al., 2016). To find an optimal combination of therapies and, putatively, a cost-efficient treatment regime, we studied possible synergistic effects between ILAT and RH rTMS. We combined them in an RCT of chronic post-stroke aphasia rehabilitation. To determine the effects of ILAT and RH rTMS on language recovery separately and in conjunction, the RCT combined these factors orthogonally. The two groups received either rTMS or sham stimulation during a 4-week period together with language training. In the first 2 weeks (T1–T2), low-intensity naming exercises were given, and in the second 2-week interval (T2–T3), high-intensity ILAT was delivered. A follow-up examination at 3 months after therapy completed the study. Therefore, this study design enables examination of effects of ILAT and TMS separately as well as any interaction between these factors. In particular, an improvement specifically in the rTMS group during the first interval (T1–T2) would support the efficacy of rTMS, and an improvement in the sham group during the second interval (T2–T3) would support an effect of ILAT. An additional hypothesis was that the combination of ILAT and TMS produces the most effective alleviation of aphasic symptoms, thus predicting the relatively strongest improvements in the second interval in the rTMS group. Therefore, our research questions were: (1) Are ILAT alone and RH rTMS alone effective therapy methods in chronic post-stroke aphasia? and (2) Is aphasia rehabilitation most effective when TMS is combined with ILAT?

## Materials and Methods

### Participants

**Figure 1:**
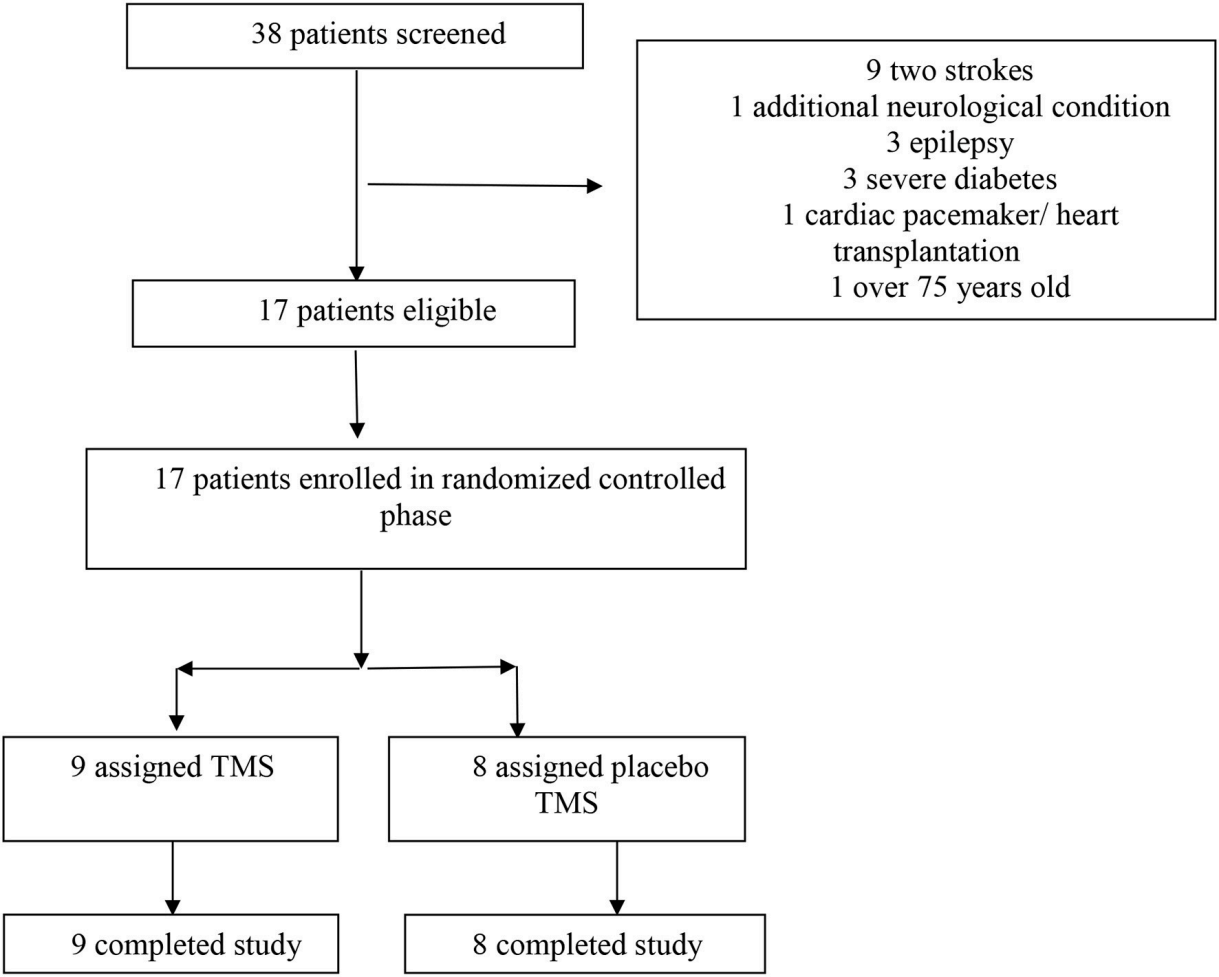
The flow of participants in the randomized, placebo-controlled study.

After screening of 38 patients, 17 patients with a neurological diagnosis of chronic post-stroke aphasia were selected to participate in this study based on pre-defined inclusion and exclusion criteria (see Figure 1 and Table 2). All the selected patients completed the study. Table 1 shows baseline characteristics and treatment randomization. At baseline, no significant group differences (groups *A* and *B*) were discovered for age, laterality, post-onset time or diagnosis. The mean AQ, however, was higher in group *B* than in group *A*. To balance the groups and to confirm the results, statistical analysis was therefore repeated for a subgroup of patients from which two outliers (subject 5 and 17) were removed. Table 2 shows clinical and sociodemographic information about the patient sample.

**Table 1:** Baseline Characteristics of the two patient groups undergoing treatment.

**Variable**	**Group *A*: rTMS (*n* = 9)**	**Group *B*: Placebo rTMS (*n* = 8)**	***p*-values between groups**
Mean age (range), year	54 (37–73) (sd:9.94)	61 (50–69) (sd:7.47)	0.093/0.189
Male, *n* (%)	7 (77%)	6 (75%)
Right handedness, *n* (%)	9 (100%)	8 (100%)
Mean education, year	12	13
Mean time since onset of aphasia (range), months	34 (11–60) (sd:490.77)	48 (15–96) (sd:881.69)	0.541/1.000
Mean WAB-AQ (range) (max. = 100)	63.2 (37–74.1) (sd:12.66)	77.8 (58.7–90.5) (sd:11.00)	0.046*/0.152
Mean BNT (max. = 60)	29 (4–53) (sd:14.76)	41 (16–58) (sd:16.24)	0.118
Mean ANT (max. = 60)	26 (4–49) (sd:13.12)	52 (5–54) (sd:18.30)	0.262

*The right column indicates significance of differences between groups for the full groups and the matched groups after removal of two outliers*.

Sd, standard deviation. *Statistically significant p-value.

**Table 2:** Clinical and sociodemographic information about the patient sample.

**Patient**	**Sex**	**Age**	**Duration of aphasia (years)**	**Etiology**	**WAB-baseline Aphasia type (AQ, max 100)**	**Lesion location**
**GROUP** ***A***. **TMS**						
1	M	53	2.9	Ischemic stroke	Conduction (70.0)	TP
2	M	49	5.1	Ischemic stroke	Anomic (74.0)	FTP, BG, INS
3	M	53	2.8	Ischemic stroke	Transcortical motor (55.8)	FTP, BG, INS
4	F	62	2.8	Ischemic stroke	Conduction (61.7)	FTP, BG, PVWM, IC INS
5	M	52	2.0	Ischemic stroke	Broca (37.0)	FTP
6	M	72	4.4	Ischemic stroke	Broca (52.5)	FP, INS, PVWM, BG, IC
7	M	37	1.0	Ischemic stroke	Conduction (72.2)	TP
8	F	58	3.6	Hemorrhage	Anomic (74.1)	BG, INS, PVWM, IC
9	M	47	1.4	Hemorrhage	Anomic (71.1)	BG, PVWM, IC
**GROUP** ***B*****. SHAM STIMULATION**						
10	M	59	4.6	Hemorrhage	Conduction (68.8)	TP
11	M	69	2.5	Ischemic stroke	Broca (71.0)	FTP, INS
12	M	50	2.7	Ischemic stroke	Anomic (87.0)	TP
13	F	54	8.2	Ischemic stroke	Anomic (80.7)	BG, PVWM, INS
14	F	63	4.3	Ischemic stroke	Anomic (87.8)	FP
15	M	68	1.3	Ischemic stroke	Anomic (77.6)	FP, PVWM, BG
16	M	54	2.0	Ischemic stroke	Conduction (58.7)	FTP
17	M	69	6.8	Ischemic stroke	Anomic (90.5)	INS, PVWM

*All lesions were in the left hemisphere. TP, temporoparietal; FTP, frontotemporoparietal, FP, frontoparietal; BG, basal ganglia; PVWM, periventricular white matter; INS, insula; IC, internal capsule*.

Subjects were recruited through local hospitals, rehabilitation clinics and aphasia support groups. Inclusion criteria were: (1) age between 18 and 75 years, (2) presence of a single clinically documented stroke, (3) chronic stage (at least 12 months post-stroke), (4) aphasia documented using the WAB test, (5) no recurring utterances or severe global (total) aphasia (Boston naming test over 3 points), (6) residual ability to understand simple task instructions, (7) availability of information about medication, (8) no neglect, agnosia, severe vision impairment or hearing loss, (9) no severe attention or memory deficits that would prohibit participation in language games, (10) apraxia diagnosed, (11) right-handedness and no left-handedness in first-order relatives, (12) native speakers of Finnish, (13) no cardiac pacemaker or other stimulators (magnetic resonance imaging, (MRI) scans are not possible; risk factors for TMS), (14) no diagnosis of severe diabetes or severe depression, (15) no additional neurological diagnoses and (16) no other interventions in the same time period, and no on-going speech therapy during the research period (includes control measurements). All candidates known to fulfill the inclusion criteria were considered. A neurologist and speech and language therapist examined the patient documents, and the patients (or significant others) were interviewed by phone. Structural MRI and tractography were recorded from all candidates who fulfilled the inclusion criteria. When MRI scans or medical information did not reveal any reasons for exclusion, candidates went through a further language assessment. Candidates who passed the language assessment criteria (criteria numbers 4, 5, 6) were included in the project.

The study was conducted in accordance with the Declaration of Helsinki, and the protocol and its amendments were approved by the Local Ethics Committee for Clinical trials (the Hospital District of Helsinki and Uusimaa). Written informed consent was obtained from each subject or significant other.

### rTMS and Naming Therapy

Magnetic stimulation was delivered with a figure-of-eight cooled coil (diameter 70 mm) of Navigated Brain Stimulation System 4 combined with the speech module (Nexstim Plc, Helsinki, Finland). The primary targeted area was the right pars triangularis. In each session, rTMS was delivered at 1 Hz for 20 min, resulting in 1200 pulses. The maximum stimulation intensity was 90% of the motor threshold (MT) determined for the left first dorsal interosseus muscle with the method of Rossini et al. (1994). The range of the MT values was 26–68% of the maximum stimulator output; this intensity produced electric fields ranging from 38 to 90 V/m (mean 55 V/m) at the targeted cortex (Supplement 1). The stimulation parameters were in accordance with international safety guidelines and regulations (Wassermann, 1998; Rossi et al., 2009); similar parameters have been used and recommended in previous studies of aphasia therapy (Naeser et al., 2005; Martin et al., 2009a,b; Barwood et al., 2011).

During the rTMS treatment, subjects named pictures (selected according to their frequency in Finnish language) displayed on the screen of the Nexstim speech module to increase the speech-related activation. Object pictures (nouns) were displayed during the first 10 min and action pictures (verbs) during the last 10 min. The pictures were presented once every 10 s. The treatment sessions were videotaped. Sham rTMS was delivered by attaching a 75-mm nonconductive plastic block (Nexstim Spacing Part) on the coil to increase the coil-to-scalp distance and minimize the electric field. This way, a similar sound could be produced as during the real TMS and a touch which corresponded that perceived when coil is placed on the scalp. The cortical electric field values (Supplement 1) for placebo stimulation were estimated to be 4–8 V/m (mean 5 V/m). Moreover, as the subjects had not received rTMS previously, they could not know whether they were receiving real or sham rTMS.

### Intensive Language-Action Therapy (ILAT)

A clinically experienced aphasia therapist delivered ILAT speech language therapy (Pulvermüller and Berthier, 2008). ILAT includes communicative language games played in an interactive small-group setting. Three persons with aphasia and one therapist sat around a table and had picture cards in front of them. Participants could only see their own card sets, as barriers blocked the view on the co-players' cards. The card sets included two items of each object or action picture. All participants took turns in making verbal requests. First, one player picked a card from their own set and then made a request to obtain the corresponding card from one of their co-players. Participants were free in choosing their linguistic tools for requesting. Apart from training requests, ILAT targets the participants' abilities to respond to requests by rejecting the request (“Oh, I do not have that”) or by repairs (e.g., asking back), along with the understanding of these speech acts. The intervention provides the opportunity to engage in a communicative interaction in accordance with turn-taking conventions and general principles of conversation. The treatment set (stimulus items) comprised over 900 cards depicting high- and low-frequency objects, colors, numbers, and objects namable by phonological minimal pairs and actions. The target vocabulary consisted of 555 nouns and 421 verbs. The original German ILAT treatment materials (kops.uni-konstanz.de/handle/123456789/11296; Neininger, 2002) were translated and adjusted to the Finnish language and culture; their feasibility for the interactive game had previously been piloted. The patients were engaged in ILAT for 3 h daily, with a short break between sessions; altogether 30 h of ILAT in 10 days (5 days a week within 2 weeks) was delivered. ILAT treatment was conducted during the same day, immediately after rTMS or sham stimulation. The therapist responsible for ILAT was blinded to the rTMS/placebo-rTMS-treatment schedule. The ILAT sessions were videotaped.

### Study Design

**Figure 2:**
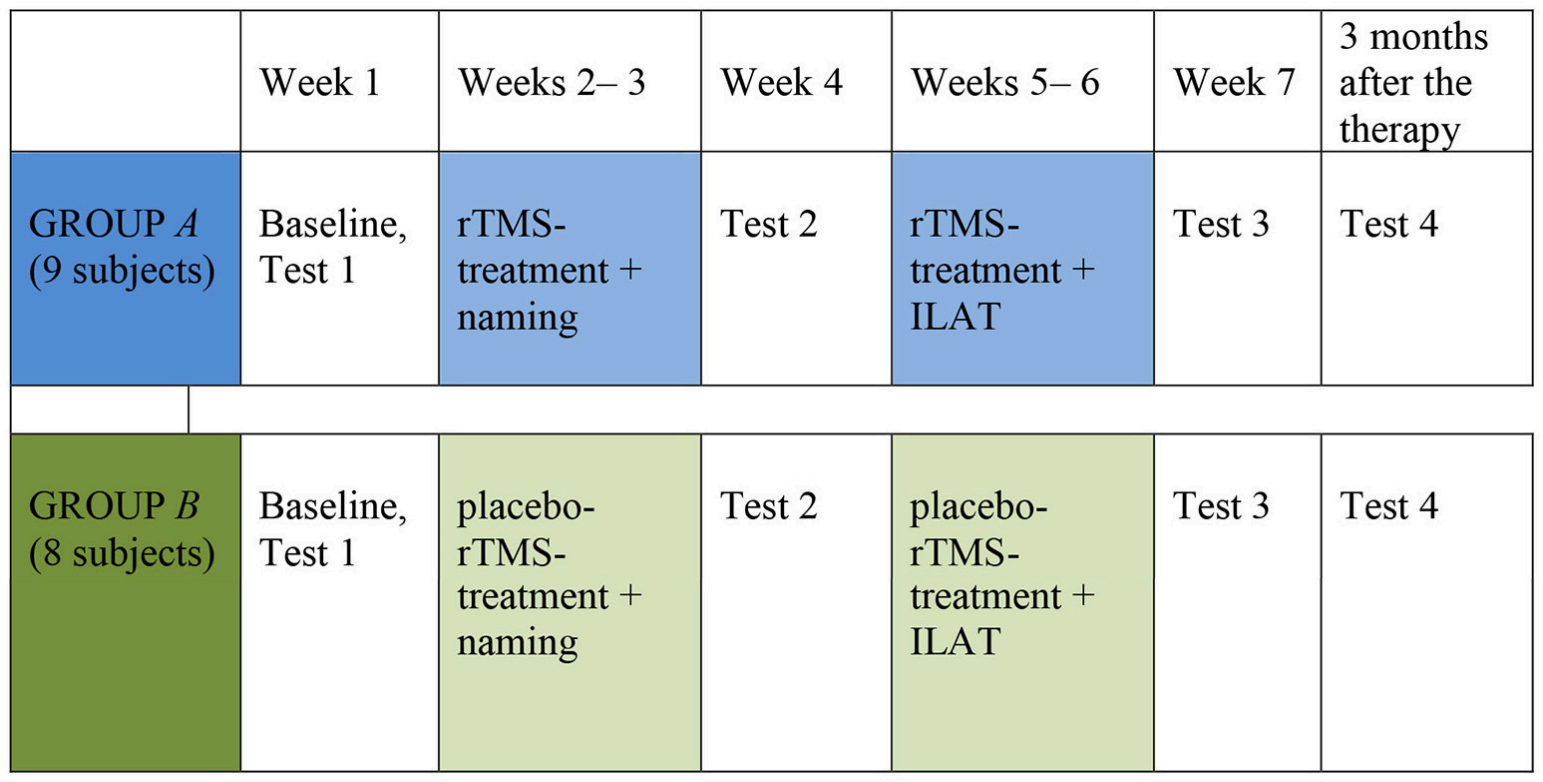
Study design.

The study design was randomized, placebo-controlled, parallel-group study of sham stimulation vs. RH rTMS + naming first given alone and subsequently combined with ILAT. The patients were divided into groups of three according to (1) similarities in aphasia characteristics (e.g., verbal output and comprehension) and (2) severity of aphasia. The groups of three patients were then randomized by throwing a dice by a person not involved in therapy, either into the intervention group *A* or into the sham group *B*. If the group was randomized to be an intervention group *A*, the following group was treated as group *B* and vice versa to ensure that equal number of subjects would be in the rTMS group and in the sham group. Group *A* received 2 weeks of rTMS followed by a 2-week period of rTMS combined with ILAT. Group *B* received a 2-week interval of sham rTMS followed by a 2-week treatment with sham rTMS combined with ILAT. The total treatment period was 4 weeks. Patients received daily rTMS (TMS group) or sham rTMS treatment (placebo group) 5 days a week for 4 weeks (altogether 20 treatments; Figure 2). The data were collected between February 2012 and February 2014.

### Outcome Measures

The primary outcome measure was the score change from baseline to the end points in the Aphasia Quotient (AQ) of the Western Aphasia Battery (WAB) (Kertez, 1982; Pietilä et al., 2005). In addition, the Boston naming test (BNT) (Kaplan et al., 1983) and the Action naming test (ANT) (Obler and Albert, 1986) were used as secondary outcome measures. All tests were performed at weeks 1, 4 and 7 and 3 months after therapy completion. The WAB-AQ is the summary score that indicates the overall severity of aphasia, and it results from the sum of subtests of spontaneous speech (fluency and information content), auditory comprehension, repetition and naming. The BNT is an assessment tool to measure confrontational word retrieval of 60 object pictures. The ANT is a confrontation naming test for 60 action pictures. The assessors were blinded to the patients' group assignments and types of intervention (i.e., the raters did not know which combination of treatment was conducted in the respective subject).

### Statistical Analysis

Data analysis was performed with SPSS for Windows (IBM Inc.). To compare age, duration of aphasia and aphasia severity scores of the two groups, a nonparametric Mann–Whitney *U*-test was performed (2 independent samples comparison). Because of the sample size, nonparametric tests were used whenever possible. Wilcoxon matched pairs test (exact significance, 2-tailed) was done to compare the language test (WAB) results before and after each intervention point. Because we had a repeated measures study design, also the time was an important factor concerning rehabilitative effect from timepoint 1 to timepoint 4. However, suitable nonparametric tests were not available to reveal the time effect and, therefore, analysis of variance (ANOVA) was used. To examine the effects of interventions on treatment outcome, a repeated-measures of variance (ANOVA) was performed with language test scores and time (T1, T2, T3, T4) as factors (independent variable). In addition, a one-way repeated measures analysis of variance ANOVA was conducted to evaluate the baseline values vs. those at other time points. Mauchly's test of Sphericity was made and effect sizes (Partial Eta Squared = η^2^) were calculated for significant results. When sphericity assumption was not fulfilled, a Greenhouse–Geisser correction determined the statistical significance. *Post-hoc* tests were done using Bonferroni correction. All statistical tests were interpreted with significance level set at α < 0.05. To explore the time effect more thoroughly, a pairwise comparison was made to determine the significance of differences between the individual time points. The statistical analysis was made first for the whole group. Thereafter, the analyses were repeated for group without outliers (subject 5 and 17), minimizing the differences between the aphasia severity levels (WAB-AQ).

## Results

### Comparability of the Groups

Subjects of both groups were comparable regarding age [U](18.000), *p* = 0.093, duration of aphasia/post-on-set time [U](29.000), *p* = 0.541 and etiology (ischemic stroke vs. hemorrhage) [U](32.500), *p* = 0.743. All subjects had the lesion in the left hemisphere. The random assignment resulted in a statistically significant difference in the aphasia severity scores between the groups. The mean aphasia quotient (WAB) was higher in group *B* [U](15.000), *p* = 0.046. Therefore, after removing two outliers and aphasia severity was comparable between groups, we performed a second set of analyses for the remaining 15 (8+7) subjects. Thereafter, the group difference of WAB-AQs had vanished ([U](15.000), *p* = 0.152). The comparisons of age [U](16.000), *p* = 0.189, duration of aphasia/post-on-set time [U](28.000), *p* = 1.000 and etiology (ischemic stroke vs. hemorrhage) [U](25.000), *p* = 0.779 remained insignificant.

### Treatment Effects

**Figure 3:**
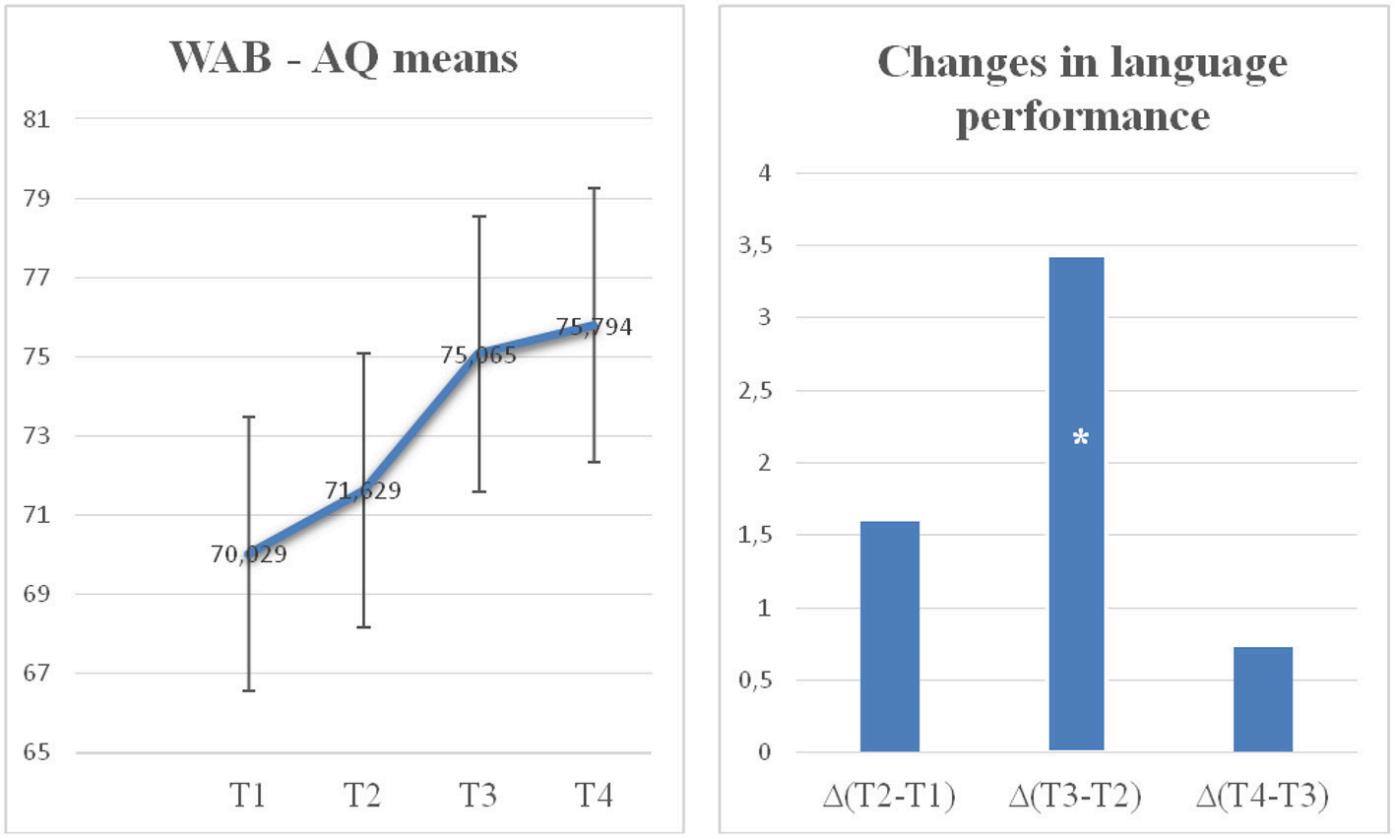
Average aphasia severity for the entire group of 17 post-stroke aphasia patients across the 4 assessments. The panel on the left shows WAB-AQ scores at timepoints T1 (baseline), T2 (after rTMS/placebo + low-intensity naming therapy), T3 (after rTMS/placebo + ILAT) and T4 (3 m follow-up). Error bars indicate standard errors of the mean. The right panel shows the changes of WAB-AQ scores across the first (T1–T2) and second therapy intervals (T2–T3) and the 3 months after therapy (T3–T4). Asterisks (*) index significant effect.

Across therapies, aphasia severity diminished in both groups. A significant improvement over time from baseline in the primary outcome measure WAB-AQ (see Figures 3A,B), and the secondary outcome measures BNT and ANT scores to follow up measurements was observed. Sphericity assumption was fulfilled in WAB (Mauchly's W = 0.659, *p* = 0.335) and BNT (Mauchlys W = 0.660, *p* = 0.337). In the interpretation of ANT (Mauchly's W = 0.291, *p* = 0.005), Greenhouse–Geisser correction was applied. The results of the ANOVA confirmed a significant time effect for both groups [WAB *F*_(1, 15)_ = 23.969; *p* = 0.001 η^2^ = 0.615; BNT *F*_(1, 15)_ = 12.350; *p* = 0.000, η^2^ = 0.452] showing that both groups improved over time. No main group effect or interaction was found. A significant main time effect across groups, without group main effect or interaction, was also found in ANT [ANT *F*_(1, 15)_ = 10.436; *p* = 0.001, η^2^ = 0.410]. Thus, both rTMS and placebo groups benefited from the therapy in a similar way.

To directly address the main hypothesis about the efficacy of ILAT, the Time effect on the WAB-AQ was calculated specifically for the interval between T2 and T3, where ILAT was given. According to the nonparametric Wilcoxon matched pairs test all participants benefited from ILAT [WAB AQ: *p* = 0.001 (exact significance, 2-tailed)]. According to ANOVA this comparison yielded a significant difference (WAB AQ: *p* = 0.006), thus confirming ILAT-induced language improvements. No effect of the Group factor was observed nor an interaction involving this factor. In contrast, the other intervals, the early rTMS interval (T1–T2: Wilcoxon matched pairs for group *A p* = 0.570, group *B p* = 0.195 and ANOVA for all participants *p* = 0.652) and the post-therapy period (T3–T4: *p* = 1.000), did not reveal any significant change in WAB-AQ. Furthermore, significantly higher AQ values were also obtained after ILAT intervention (T3) compared with study onset (time points T1, WAB-AQ: *p* < 0.001). The change relative to the baseline remained significant during the follow-up period (T1 vs. T4, WAB-AQ: *p* < 0.001 T2 vs. T4, WAB-AQ: *p* < 0.001), thus demonstrating a lasting improvement. The statistically significant change between time points T2 and T3, but not in other intervals, documents a specific effect of ILAT in improving clinical language profiles in chronic aphasia.

The specific rTMS hypothesis is that the rTMS group, but not the sham stimulation group, shows a significant improvement of language performance between T1 and T2. However, the absence of a significant interaction of the Group and Time factors failed to provide strong support for this hypothesis. Furthermore, neither Group *A* nor Group *B* provided any evidence for significant performance change during this first therapy interval. Numerically, the average amount of change seen during TMS stimulation with picture naming was 1.5 WAB-AQ points in the rTMS group, whereas that in the sham/placebo group was 1.7. These results do not support the idea that a difference might have emerged with greater statistical power. Comparisons across the entire period during which TMS vs. sham stimulation were applied revealed comparable and significant improvements, which did not differ between groups (amount of change: 5.7 vs. 3.2 WAB-AQ points). Across all four time intervals, there was again no significant difference between the sham and TMS groups (mean differences 5.6 vs. 5.8 WAB-AQ points). Furthermore, the interactions of the factors Group (*A* vs. *B*) × Time (T1 vs. T2 and T1 vs. T3) were far from significant on all outcome measures applied. Our study thus provides no support for the effects of 1-Hz rTMS to the right pars triangularis in chronic post-stroke aphasia.

Because the baseline WAB-AQ scores differed between groups *A* and *B*, a secondary statistical analysis was performed after outlier removal (*n* = 15). This analysis confirmed all results obtained from the whole group. The results of the ANOVA indicated a significant time effect for both groups [WAB *F*_(1, 13)_ = 23.254; *p* = 0.000, η^2^ = 0.641], whereas no group effect or interaction was found. Pairwise comparisons showed significant improvements after ILAT, in the comparisons between time points T1 (baseline) and T3 (WAB AQ: *p* = 0.001) and T2 and T3 (WAB AQ: *p* = 0.03). The change was significant also during the follow-up period T1 and T4 (WAB-AQ: *p* < 0.001) as well as T2 and T4 (WAB-AQ: *p* = 0.001). In contrast, the other intervals, the early rTMS interval (T1–T2: *p* = 0.241) and the post-therapy period (T3–T4: *p* = 0.706), did not reveal any significant change in WAB-AQ. These results, confirmed the earlier timepoint analysis result for the whole group (*n* = 17), suggesting that participation in the ILAT treatment increased the level of linguistic performance.

Because WAB is a combination of subtests, we analyzed also the improvement in those separately. Subjects of both treatment groups improved in all other subtests of the WAB except comprehension. For the group of all participants (*n* = 17) the sphericity assumption was fulfilled in WAB Comprehension (Mauchly's *W* = 0.654, *p* = 0.325) and WAB Naming (Mauchly's W = 0.594, *p* = 0.210). In the interpretation of WAB Spontaneous speech (Mauchly's W = 0.383, *p* = 0.022) and WAB Repetition (Mauchly's W = 0.366, *p* = 0.017), a Greenhouse–Geisser correction was used. The results of the ANOVA indicated a significant time effect (showing that both groups improved) across both groups in all subtests except comprehension [TIME:WAB Spontaeous speech *F*_(1, 15)_ = 15.758; *p* = 0.000, η^2^ = 0.512; Comprehension *F*_(1, 15)_ = 1.932; *p* = 0.138, η^2^ = 0.114; Repetition *F*_(1, 15)_ = 3.346; *p* = 0.027, η^2^ = 0.182; Naming *F*_(1, 15)_ = 14.538; *p* = 0.000, η^2^ = 0.492]. There was no difference between which one of the groups the subject belonged to [GROUP:WAB Spontaneous speech *F*_(1, 15)_ = 0.608; *p* = 0.613, η^2^ = 0.039; Comprehension *F*_(1, 15)_ = 0.645; *p* = 0.590, η^2^ = 0.041; Repetition *F*_(1, 15)_ = 0.457; *p* = 0.714, η^2^ = 0.030; Naming *F*_(1, 15)_ = 1.353; *p* = 0.269, η^2^ = 0.083]. When statistical analysis was made after outlier removal in baseline-balanced groups (*n* = 15) the only difference to the above results was that even comprehension showed statistically significant improvement for both of the groups [TIME:WAB Comprehension *F*_(1, 13)_ = 3.092; *p* = 0.038, η^2^ = 0.192].

## Discussion

With the present RCT, we aimed at finding an optimal protocol for the neurorehabilitation of chronic post-stroke aphasia by combining two promising methods, ILAT (Intensive language action therapy) and 1-Hz rTMS to the right-hemispheric homolog of the anterior language area of Broca (pars triangularis), thus taking advantage of recent research in speech and language sciences, neurorehabilitation and brain research. TMS was used in a clinically applicable way. Seventeen participants were randomized into two groups; one group receiving rTMS, the other sham stimulation. This was accompanied by a low-intensity naming training during the first 2 weeks, followed by 2 weeks of ILAT delivered 3 h per day. Whereas our results confirm that ILAT therapy has a beneficial effect on language performance in chronic post-stroke aphasia, we found no evidence for an effect of rTMS. Likewise, the hypothesis of a synergistic effect of RH rTMS and ILAT could not be confirmed by our present data.

### Efficacy of High-Intensity, Communication-Embedded and Patient-Tailored ILAT

During the delivery of ILAT, the scores of the Aphasia Quotient calculated from the Western Aphasia Battery (WAB-AQ), as well as those of our secondary measures, the object (BNT) and action naming tests (ANT), improved equally in both groups. This finding is in line with the previous studies applying this method (Pulvermüller et al., 2001; Meinzer et al., 2005; Berthier et al., 2009; Szaflarski et al., 2015; Stahl et al., 2016, 2018; Mohr et al., 2017). These studies have shown that within a short period of time (typically 2 weeks), language performance can be improved at the chronic stage of post-stroke aphasia by highly intensive language training embedded into social communication and constrained to match the patients' individual communicative needs.

Our study design enabled interpretation of the statistically significant training effect as a specific effect of ILAT. When analyzing the pairwise comparisons of successive time points across the interventions, the only statistically significant change occurred between time points T2 and T3 across the time period where ILAT was delivered. This change documents a specific effect of ILAT in improving clinical language profiles in chronic aphasia. As a significant difference was present for the comparison of time points T2 and T3, but not in the interval where TMS/sham stimulation were paired with low-intensity naming (T1–T2) or in the 3 months following therapy (T3–T4), our data speak strongly in favor of the efficacy of ILAT. The follow-up measurement indicated a stable effect lasting for months. Furthermore, because both rTMS and sham stimulation groups improved in the second therapy interval, the negative result regarding rTMS cannot be due to general unresponsiveness to interventions of the rTMS group. Such unresponsiveness would only have been plausible in case of an overall absence of treatment effects in this group.

ILAT has proven to be efficient especially for two reasons; because of its intensity and its communicative and social-interactive aspect. Apart from its high intensity with (up to) 30 h of practice in less than 2 weeks, ILAT emphasizes the training of language skills in the context of communication and social interaction (Difrancesco et al., 2012). Although some work indicates that high therapy frequency alone may be sufficient for achieving good progress (Bhogal et al., 2003), some recent evidence suggests communicative embedding as a further relevant feature. Stahl et al. (2016) compared ILAT with an equally intensive and exactly matched confrontation naming therapy conducted with the same pictorial and linguistic materials as ILAT. In intensive naming therapy, the focus was on speech production and language comprehension *per se*, which contrasted with the ILAT-specific embedding of language into communication purposes and social interaction. The study by Stahl et al. (2016) showed that ILAT was generally more beneficial than intensive naming in chronic non-fluent aphasia. Confrontation naming also led to positive rehabilitative outcomes but only if it was given before ILAT and to a lesser degree than ILAT. After ILAT, it showed no statistically significant effect. This result supports the relevance of the ILAT principle of behavioral relevance and communicative embedding. The necessity of constraints to focus patients on verbal language (instead of non-verbal gesturing, facial expression etc.) has recently been questioned. A study by Kurland et al. (2016) contrasted the ILAT method with its unconstrained version allowing free choice of communication channels (as in PACE therapy, see Davis and Wilcox, 1985); this work indicated that constrained and unconstrained versions of ILAT are equally efficient in improving chronic post-stroke aphasia.

Because no reliable effects of rTMS were present after the first therapy interval, this first interval provides a baseline against which the effect of ILAT can be compared. Because this “baseline” involved both low-intensity verbal activation i.e. naming and a highly technical device on the influences of which patients might have high expectations, it is a relatively perfect placebo control. Comparison against such a baseline is more meaningful than no baseline at all or an interval during which patients just wait for the onset of intervention (e.g., Breitenstein et al., 2017). Waiting for therapy may simply induce higher expectancies in patients, thus possibly inducing an even greater placebo effect later on. The non-significant profile in the present baseline (T1–T2) together with the subsequent improvement during ILAT (T2–T3) argue strongly for a genuine effect of ILAT over and above any placebo mechanisms.

### Efficacy of RH rTMS Stimulation and Naming Therapy

Somewhat in contrast with the conclusions from of a recent meta-analysis (Kapoor, 2017), we could not show the effectiveness of right-hemispheric rTMS at the group level. The lack of rTMS effects may relate to specific features of the methods applied in our study. rTMS protocols vary across studies, although most studies, including ours, have used 1-Hz rTMS to the right pars triangularis, which is homotopic to the anterior part of the language area of Broca (Mendoza et al., 2016). It is unlikely that lesion location or stimulation intensity explain the lack of therapeutic effect in our study. We stimulated the right pars triangularis, as a range of previous studies did. Some studies tried to individually locate a specific anterior-inferiorfrontal focus by using the patients' language performance under TMS as a criterion (e.g., Naeser et al., 2005). However, such a time-consuming and taxing procedure is difficult to perform under clinical conditions, which somewhat contrasts with the urge to bring neurostimulation methods to practical use (Raymer et al., 2008; Kleim, 2011). Also, the stimulation parameters used in our study (frequency, strength and duration of stimulation) were similar to those of previous studies. 1-Hz rTMS is a standard in aphasia studies as is the stimulation intensity of 90% of the individually determined motor threshold. One may argue that stimulus intensity volumes (exact V/m values) were not usually reported precisely per subject in the previous studies (e.g., Barwood et al., 2011; Medina et al., 2012; Khedr et al., 2014), so that a difference in absolute stimulation intensities may have been present between previous and our present study. Still, comparing the values reported in those studies as reported (i.e., 90% of the motor threshold MT), stimulus intensities were matched and thus unlike to provide an explanation.

The average MT in group *A* was 38%. It is slightly smaller than that described in some previous stroke studies [e.g., 51% in Bütefisch et al. (2003); 50% (range 32–70) in Blicher et al. (2009)] It should be noted, however, that the devices differ; Butefisch et al. and Blicher et al. used a Magstim 200 stimulator, and we had a navigated Nexstim device at our disposal. With Nexstim stimulator using biphasic pulses, the MT in healthy subjects is 43 ± 9% of the stimulator output (Danner et al., 2012). The Nexstim software enables estimation of the induced cortical electric field; the average value in our patients was 55 V/m. This exceeds the value needed to activate the cortex in TMS-EEG measurements (Casali et al., 2010), and is in line with 52 ± 10 V/m field strength estimated as MT of biphasic Nexstim stimulation in the depth of 25 mm (Danner et al., 2012). In some patients, cortical excitability is increased in the non-lesioned hemisphere probably due to lack of inhibitory activity from the lesioned side. This may explain the larger variability of cortical excitability in the non-lesioned hemisphere of stroke patients. The MTs are typically tested for single pulse stimulations, whereas we delivered a 1-Hz stimulation train for 20 min. It is highly probable that this type of stimulus cumulatively activates the underlying cortex.

A main difference in the stimulation protocol exists between this and previous studies with regard to the timing of behavioral speech language therapy and rTMS. In recently published studies addressing post-stroke aphasia, TMS was given immediately before speech and language therapy (e.g., Mendoza et al., 2016; Kapoor, 2017). The type, duration and intensity of speech language therapy following the stimulation varies across studies. The systematic review of Mendoza et al. (2016) included 15 articles. In 10 of them TMS was followed by speech language therapy. For example, in the study of Khedr et al. (2014), rTMS was followed by 30 min of speech and language training, using subtests of the Boston Diagnostic Aphasia Examination as training material. In the study of Tsai et al. (2014), rTMS was followed by 1 h of speech therapy emphasizing expressive production including semantic training, phonemic training, repetition naming, conversation, picture-description tasks, and phrase generation tasks. Moreover, participants engaged in home exercises for 30 min daily, including mainly picture naming tasks. We chose to combine rTMS with simultaneous naming exercises. This innovative element was introduced to increase the TMS effect by activating the language-related areas simultaneously with rTMS stimulation. A motivation for this strategy comes from rTMS research of motor function. Simultaneous behavioral exercise and TMS stimulation has proved successful in the modification of motor function (Jochumsen et al., 2016). Different timing of TMS and behavioral exercises may, however, be adequate in motor (simultaneous application) and language therapy (sequential application). Evidently, these issues require further research.

Word-finding difficulties are common in all types of aphasia; anomia is one of the most persistent features in all residual cases of aphasia. There are several kinds of therapy approaches to rehabilitate anomia (Wisenburn and Mahoney, 2009). In the first therapy interval between T1 and T2, we used confrontation naming as a supplement to rTMS and sham stimulation; the patients named object and action pictures during rTMS. The subjects did not get any help or cues during the procedure; no assistance nor feedback, was provided for the participants as to the correctness of the response to the exposed word set. If they failed to name a picture, it just went pass and the next picture was presented automatically. This type of naming training did not prove to be efficient in our study. The confrontational naming was included to all rTMS sessions (20 min 5 times a week during 2 weeks). This cannot be considered an intensive therapy, as intensity remained below the 5 h-per-week threshold commonly assumed for intensive treatment (Bhogal et al., 2003). Also, confrontation naming did not contain any communicative aspects, a fact which may in part explain why no evidence for related improvement was obtained. Stahl et al. (2016) argue that in order to make aphasia therapy efficient, it is necessary to use language as a tool for communication and social interaction. Furthermore, the findings of a meta-analysis (Wisenburn and Mahoney, 2009) showed that although different naming therapy approaches might be beneficial, semantic naming therapy generalized best to untrained words. Also, the low dosage of confrontational naming (20 min once per day equaling < 2 h per week) may contribute to the explanation of lacking effects. We remind that, according to Bhogal and colleagues, at least 5 h per week are required to yield improvements (Bhogal et al., 2003). Still, in our present study and similar TMS work, the purpose of the naming task was to complement and potentially enhance any rTMS-related effect, so that naming *per se* did not function as an independent intervention.

One could also argue that the good result with ILAT might be due to prolonged treatment period of 4 weeks in total (2 weeks period of low intensity naming + 2 weeks period of ILAT). Moreover, although low-intensity naming was not effective itself, it might have had a priming effect to boost the result of the ILAT period. This does not, however, rule out the effectiveness of the 2-week ILAT intervention ILAT, since the statistically significant change was observed only after ILAT intervention. Thus, our result on ILAT confirm the results of the earlier studies of ILAT being effective form of therapy in only 2 weeks (Pulvermüller et al., 2001; Meinzer et al., 2005; for review, see Meinzer et al., 2012).

The lack of strong evidence for rTMS effects in this study needs to be treated cautiously because of the small size of the patient sample (*n* = 17). We wish to point out, however, that most previous studies used even smaller sample sizes and some even missed any control group. Importantly, our study was an RCT and RCTs have only rarely been performed in the assessment of rTMS-related therapy effects on language. For example, an influential paper by Naeser et al. (2005) was based on data from four aphasics treated with rTMS. Meanwhile, several RCTs have included larger numbers of patients. In particular, Thiel et al. (2013) report results from 24 and Khedr et al. (2014) from 30 aphasic patients, randomized to TMS and sham groups. Both studies found support for a therapeutic benefit of RH rTMS in subacute stroke. Spontaneous recovery, however, varies strongly early after a stroke, so that a relatively large number of patients was needed to obtain the statistical power necessary for obtaining any significant effects. In contrast, at the chronic stage very little or no spontaneous recovery can be expected within a period of a few weeks. Consequently, the variability in such negligible improvement is low, thus implying substantially more statistical power for short-term intensive therapy in chronic stroke. A recent RCT with 12 chronic aphasia patients randomized to two groups of TMS-accompanied language therapy (*n* = 6) or sham stimulation (*n* = 6) indicated an advantage for real magnetic stimulation (Barwood et al., 2011, 2013). The TMS-related advantage, however, was not present after termination of therapy but only weeks after it, thus raising questions about possible neuroplastic effects emerging with such substantial delays. Crucially, assuming the power underlying this previous small-group RCT, our present null result in a larger population (*n* = 17 vs. *n* = 12) raises doubts in the generalizability of the earlier finding. In summary, whereas there is solid evidence for the efficacy of RH rTMS intervention at the subacute stage, the RCTs available so far do not provide clear evidence for a beneficial effect of such stimulation in chronic post-stroke aphasia.

### Limitations and Perspectives

Apart from being relatively small (*n* = 17), our patient sample was heterogeneous, with different aphasia syndromes being included. In the future, it will be necessary to investigate a larger and ideally more homogeneous group of patients (e.g., either fluent or non-fluent aphasics only; cf. Stahl et al., 2016). This may allow further reduction of variance and thus make any rTMS-related effects more likely to yield significance. We also note that our randomization procedure produced two groups that were significantly different with regard to their overall aphasia severity, as measured by the WAB-AQ. We hasten to add, however, that even after removing one severity-outlier from each group, which removed the significance of between-group differences in WAB-AQ, all results remained significant so that any group differences in overall performance are unlikely to limit the conclusions from this work. Furthermore, the real rTMS group tended to show more severe aphasia (i.e., lower scores in WAB) so that the lack of significance in progress made by this group in the first therapy interval cannot be explained by a ceiling effect. This possibility is also ruled out by the clear progress made by this group in the second therapy interval where ILAT was delivered. Real rTMS and sham stimulation led to comparably insignificant effects on WAB-AQ scores, whereas ILAT showed a significant improvement.

Our negative result of TMS does by no means imply that TMS can have no added effect to language therapy. As pointed out, limitations of our study include the relatively small sample size, the heterogeneity of the patient group, and the imbalance—or tendency toward imbalance—of the randomized groups with regard to overall aphasia severity. The efficiency of ILAT could, however, be demonstrated in spite of these limitations. Using language for communication is inherently a social activity (Stahl et al., 2016; Mohr et al., 2017). Presumably, learning processes induced by social-interactive language games implemented in ILAT are more suitable for the neurorehabilitation of human communication than stimulating directly the right-hemispheric brain areas homotopic to those areas participating in language processes, without implementing any communication context. Although we could not provide any evidence for synergistic effects between ILAT and right-hemispheric inferior-frontal rTMS, it will be important to further explore the possibility that specific cortical stimulation methods might further improve the effects of successful behavioral methods for the rehabilitation of chronic post-stroke aphasia.

According to the recent Cochrane review by Brady et al. (2016), there are significantly higher drop-out rates in high-intensity or “high-dose” therapy settings than in low-intensity or “lower-dose” therapies. Somewhat in contrast with this postulate, all our 17 patients completed the study. This converges with earlier publications on ILAT or Constraint-Induced Aphasie Therapy given to patients with chronic aphasia (e.g., Pulvermüller et al., 2001; Meinzer et al., 2005; Berthier et al., 2009; Stahl et al., 2016, 2018; Mohr et al., 2017), which, similar to the present study, did not report dropouts in spite of high therapy intensity. We note that all our subjects were highly motivated to participate in this kind of therapy and gave positive feedback about the high intensity. It is possible that the high dropout rates summarized by Brady and colleagues relate to the conjunction of therapy intensity and other factors, e.g., the application of specific therapy methods or delivery at early stages post-onset of the disease.

In conclusion, ILAT turned out to be effective at the chronic stage of post-stroke aphasia and useful in the clinical neurorehabilitation of language. This positive result can be used to further optimize evidence-based treatments for aphasia therapy. Other positive results were that there were no drop-outs, and that the subjects gave positive feedback about the high intensity of the therapy as well as the social aspect of group rehabilitation. In contrast, an RH rTMS protocol optimized for clinical applicability did not produce significant effects. Our data motivate a large-scale multi-center study of the effects of both ILAT and RH rTMS.

## Author Contributions

All authors participated in planning the study. PH, PL, AA and R-LM collected the data. PH analyzed the data. PH, FP, JM, RI, PL and AK wrote the manuscript and interpreted the data. All authors have critically read the manuscript and approved the final version of the study to be published.

### Conflict of Interest Statement

RI is an advisor and a minority shareholder of Nexstim Plc. PL has been a paid consultant for Nexstim Plc for the motor and speech mapping rTMS applications before 2017. Nexstim Plc has provided travel and accommodation expenses for lectures in conferences for JM. The remaining authors declare that the research was conducted in the absence of commercial or financial relationships that could be construed as a potential conflict of interest.
