# Supplementary material for: Combining rTMS With Intensive Language-Action Therapy in Chronic Aphasia: A Randomized Controlled Trial
Source: Front Neurosci. 2019 Feb 4;12:1036. doi: 10.3389/fnins.2018.01036 (PMC6369187; doi:10.3389/fnins.2018.01036)
Supplement: Supplementary file 1 [file Table_1.pdf]

Supplement 1. Motor thresholds and Stimulation values

**Subject**	**Motor threshold means**	**V/m means**
**Group *A***		
1	36	61
2	41	66
3	38	36
4	68	90
5	26	38
6	40	51
7	28	55
8	26	40
9	37	55
Group *A* means (rTMS)	**38**	**55**
**Group *B***		
10 placebo	39	4
11 placebo	33	4
12 placebo	38	4
13 placebo	76	8
14 placebo	36	4
15 placebo	74	8
16 placebo	35	4
17 placebo	34	5
Group *B* (placebo rTMS) means	**46**	**5**

V/m was estimated at the stimulated cortex. The values for the placebo group *B* are approximately 10% of the ones considered to excite the cortex (Casali et al., 2010).
